# Identification and Molecular Characterization of a Chitin-Binding Protein from the Beet Webworm, *Loxostege sticticalis* L.

**DOI:** 10.3390/ijms151019147

**Published:** 2014-10-22

**Authors:** Jiao Yin, Shuang Yang, Kebin Li, Wei Guo, Yazhong Cao

**Affiliations:** 1 State Key Laboratory for Biology of Plant Diseases and Insect Pests, Institute of Plant Protection, Chinese Academy of Agricultural Sciences, Beijing 100193, China; E-Mails: jyin@ippcaas.cn (J.Y.); tinyys@126.com (S.Y.); kbli@ippcaas.cn (K.L.); 2 Plant Science and Technology College, Beijing University of Agriculture, Beijing 102206, China

**Keywords:** peritrophic membrane protein, *Loxostege sticticalis*, chitin-binding protein, LstiCBP

## Abstract

As the first crucial barrier in the midgut of insects, the peritrophic membrane (PM) plays an important role in preventing external invasion. PM proteins, as the major components of the PM, determine the structure and function of this membrane. A new PM protein, named LstiCBP, from the PM of *Loxostege sticticalis* larvae was identified using cDNA library screening. The full cDNA of LstiCBP is 2606 bp in length and contains a 2403 bp ORF that encodes an 808-amino acid preprotein with a 15-amino acid as signal peptide. The deduced protein sequence of the cDNA contains 8 cysteine-rich chitin-binding domains (CBDs). Recombinant LstiCBP was successfully expressed in BL21 cells using recombinant plasmid DNA and showed high chitin-binding activity. LstiCBP expression was detected in the midgut at both the transcriptional and translational levels; however, the biochemical and physiological functions of LstiCBP in *L. sticticalis* require further investigation.

## 1. Introduction

The peritrophic membrane, a structure in the gut of insects that is unique to invertebrates, is believed to be their initial protection from invasion by viruses, bacteria, protozoa, and helminthes, and it prevents damage to midgut cells by abrasive food particles [1,2,3]. In the course of the co-evolution of microorganisms and insects, some microorganisms formed several mechanisms to destroy the PM. Meanwhile, many factors, for example, *Pseudaletia unipuncta* GV (PuGV) enhancin, chitinase, calcoflour and lectin, can disrupt the formation of the PM and enhance pathogen infection in insects [4]. Therefore, as a natural barrier to pathogenic microorganisms, the PM has become a potential target for insect control [5].

The insect PM is mainly composed of proteins and chitin, with chitin-binding activities as their typical characteristics. The identification and characterization of PM proteins from a wide variety of insects will help to develop pest management targets as well as provide a better understanding of the function and development of the PM. Currently, significant progress toward understanding the molecular structure and formation mechanism for the PM has been made, and more than 30 PM proteins or putative PM proteins have been identified from several insects [6,7,8,9,10,11,12,13,14,15,16,17,18,19,20,21,22,23,24,25,26,27]. Four classes of PM proteins have been suggested based on the solubility of the proteins under different extraction conditions [2]. Class 1 PM proteins are those that can be removed by washing with physiological buffers, Class 2 represents the PM proteins that are extractable by mild detergents, Class 3 PM proteins include those that are only extractable by strong denaturants, and Class 4 PM proteins are not extractable, even by strong denaturants. Class 3 proteins are the most abundant proteins that are extracted from PMs. These proteins usually have chitin-binding domains, or peritrophin domains. Structural characterization of PM proteins has mainly focused on the following classes: peritrophins, invertebrate intestinal mucins, and proteins with chitin deacetylase domains [2,28]. The peritrophins contain 60–75 amino acid residues and are characterized by a conserved register of cysteine residues and a number of aromatic amino acid residues [2]. The conserved cysteine residues are suggested to form intradomain disulfide bonds that contribute to protein stability in the protease-rich gut environment [2,8,9,10]. Insect Intestinal Mucin (IIM) is a highly glycosylated, mucin-like protein that binds very strongly to the type 1PMs identified in *Trichoplusiani* larvae [10,29], and it contains peritrophin-A domains. Chitin deacetylase (CDA; EC 3.5.1.41) is a hydrolytic enzyme that catalyzes the hydrolysis of the acetamido group in the *N*-acetylglucosamine units of chitin and chitosan, thus generating glucosamine units and acetic acid [30]. The CDAs were recently found as a new component of the insect PM [18,26].

The beet webworm, *Loxostege sticticalis* L. (Lepidoptera: Pyralidae), is a polyphagous pest, which can feed on 35 families and 200 species plants and crops, such as corn, bean, potato, sugar beet, sunflower and so on. It has caused severe economic damage almost every year and became one of the worst pests in Asia, Europe, and North America and [31]. In this study, we identified a new PM protein from *Loxostege sticticalis* larvae by cDNA library screening, which was named as LstiCBP. The new PM protein exhibits a strong chitin-binding activity, which allows the protein to perform its role in PM formation.

## 2. Results and Discussion

### 2.1. Cloning of the CBP cDNA of Loxostege sticticalis

**Figure 1:**
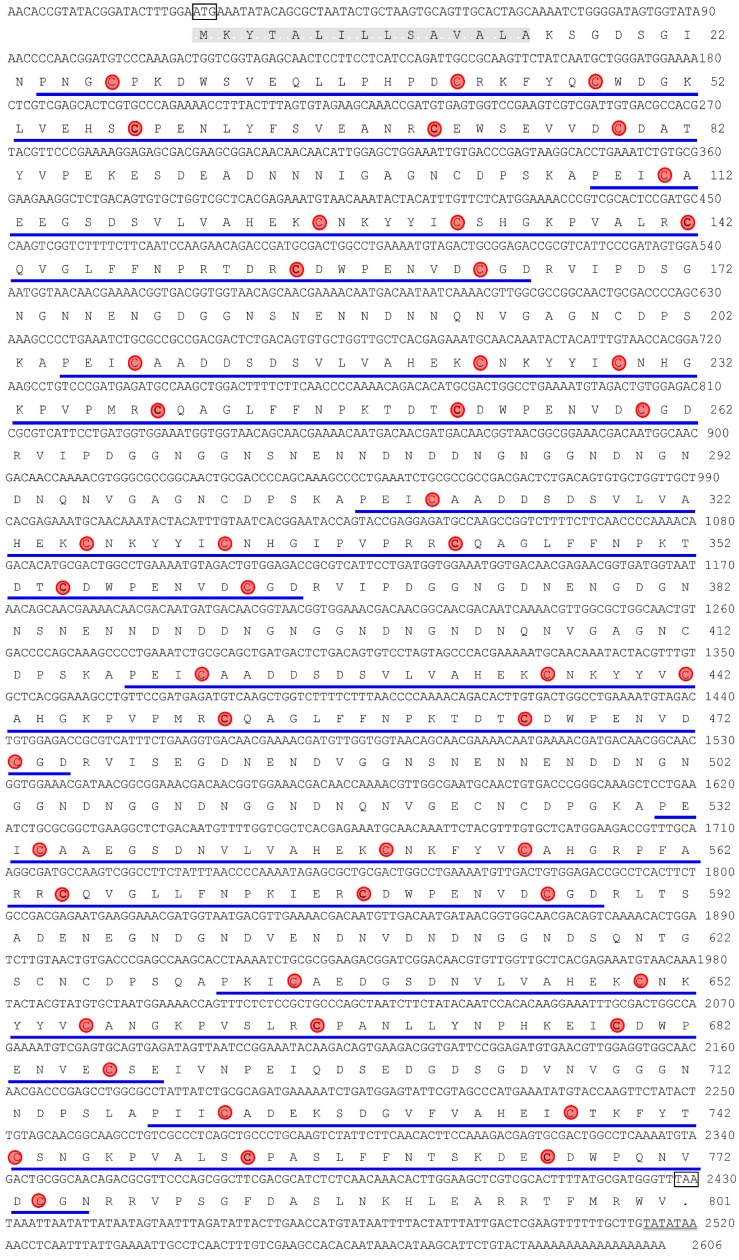
Nucleotide sequence of the cDNA for *L. sticalitis* CBP and its deduced amino acid sequence. Signal peptide domains (grey background), cysteine (red background)-rich regions (CBD1-8, underlined), the initiation and translation stop codon (in box) are indicated. The potential polyadenylation signal sequence is double lined. Eight chitin binding domains are underlined from *N*- to *C*-terminus of the protein.

Using rapid amplification of cDNA ends (RACE)-PCR, a full-length, 2606 bp cDNA encoding CBP was cloned from *L. sticticalis* (Figure 1) (GenBankFJ408730). The open reading frame (ORF) of the *Lsti*CBP cDNA consists of 2403 nucleotides and encodes a predicted precursor protein containing 801 amino acids. The ORF is terminated by a TAA stop codon that is followed by an AT-rich untranslated region and a putative polyadenylation signal (TATATAA) located at 69 bp upstream of the polyA tail (Figure 1). The deduced protein sequence revealed a 15-amino acid signal peptide, as predicted by the SignalP software. The calculated molecular weight and the isoelectricpoint of the mature LstiCBP were 84.7 kDa and 4.14, respectively. The hydrophilic nature of LstiCBP, which is similar to that of other insect CBPs [19,32], was calculated and plotted for each residue in the sequence, which revealed that eight residue regions were hydrophobic. The LstiCBP protein was analyzed to determine if it is glycosylated similar to that of IIM, and NetNGlyc 1.0 analysis showed that LstiCBP only has three *N*-glycosylations. Trypsin and chymotrypsin cleavage sites were also identified using online sequence analysis [33] which identified 55 trypsin cleavage sites and 128 chymotrypsin cleavage sites in the LstiCBP sequence.

Proteins are the primary components of the PM, and the binding of these proteins to chitin fibrils has been suggested to be important in the formation of the PM [2,8,9,10]. In this study, we identified a new PM chitin-binding protein, CBP, from *L. sticticalis* and found that cDNA clones for this protein were abundant in the non-normalized midgut cDNA expression library, which was in agreement with the previous observation that the majority of PM proteins are chitin-binding proteins. Different from invertebrate intestinal mucin (IIM), which is thought to be the most important protein of the known PM proteins, LstiCBP is not glycosylated. In Lepidopteran larvae, trypsins and chymotrypsins are the predominant digestive proteinases in the midgut. Surprisingly, the LstiCBP sequence abounds with potential trypsin and chymotrypsin cleavage sites, but the protein is highly resistant to trypsin degradation. Analysis of the trypsin and chymotrypsin cleavage sites in the sequence showed that most sites were in the chitin-binding domains. This observation is consistent with early reports showing that most trypsin and chymotrypsin cleavage sites are protected against midgut digestive proteinases by being buried in the chitin-binding domains, which is critically important because PM proteins must function in an environment that is rich in proteinases [32].

### 2.2. Characterization of LstiCBP Chitin-Binding Domains

**Figure 2:**
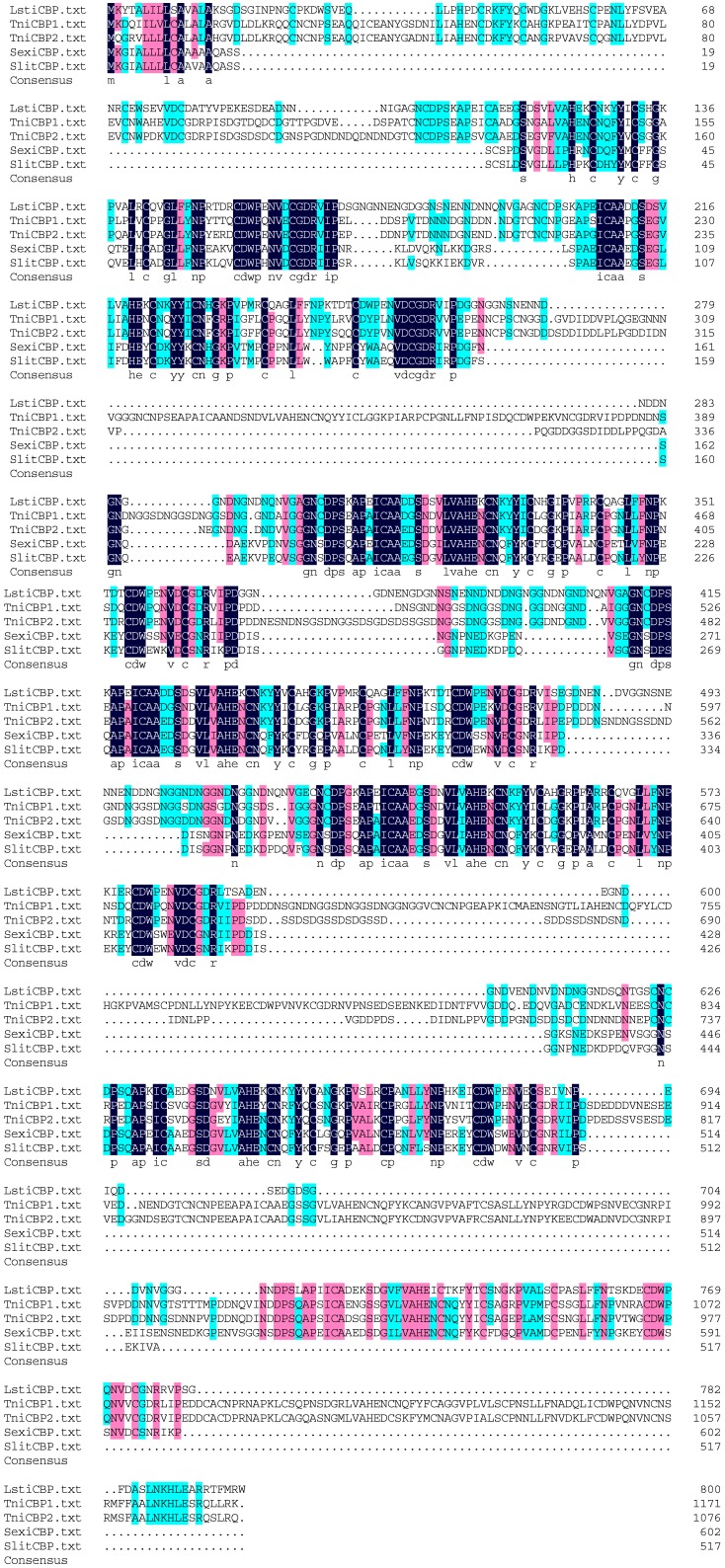
Alignment of the amino acid similarity of LstiCBP from known insects. *Trichoplusia ni* CBP1: AAR06265.1; *Trichoplusia ni* CBP2: AAR06266.1; *Spodoptera exigua* CBP: ABW98673.1; *Spodoptera litura* CBP: ADV03161.1.

**Figure 3:**
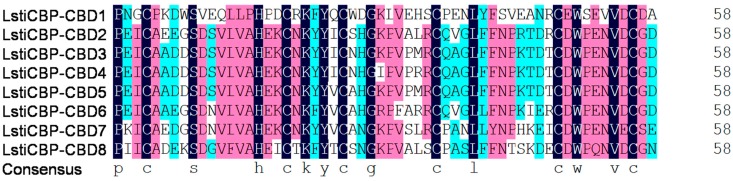
Alignment of chitin-binding domains (CBDs) from *Loxostege sticticalis* LstiCBP protein. The conserved amino acids are shaded. The consensus sequence is shown at the bottom.

The amino acid sequence alignment of LstiCBP with the known insect CBPs, TniCBP1, TniCBP2, SexiCBP, and SlitCBP is shown in Figure 2. Similar to most identified PM proteins, which contain multiple chitin-binding domains and are characterized by a conserved register of cysteine residues as well as by a number of aromatic amino acid residues [2], 8 cysteine-rich regions have been identified in LstiCBP, each of which possess a register of 6 spatially conserved cysteine residues that form a putative CBD (Figure 3). The CBDs of LstiCBP have a conserved sequence motifs, CX_14__-15_CX_5_CX_9_CX_12_CX_7_C, which is similar to the predicted chitin binding sequences of CBPs of *T. ni* and other PM proteins [2,8,9,10,20,21,32,34] and belong to the peritrophin-A domain family [2].

Early research has shown that most PM proteins have a large number of chitin-binding domains, which allows their partially degraded protein fragments to retain multiple chitin-binding domains and thus, allows their function to cross-link chitin fibrils during PM formation in the extremely six-cysteine motifs that belong to the peritrophin-A domain family (Figure 3). Like CBP1 and proteinase-rich environment [32]. In this study, LstiCBP was found to have 8 tandem, conserved, CBP2 of *T. ni*. [32], the presence of 8 chitin-binding domains in the proteins could allow for the partially degraded protein fragments to retain multiple chitin-binding domains and thus retain their functions in PM formation. On the other hand, the observations that (1) the trypsin and chymotrypsin cleavage sites in CBP are mostly located within the chitin-binding domains and (2) the proteins are composed of numerous chitin-binding domains, indicated a mechanism for these non-mucin PM proteins to adapt and function in the proteinase-rich gut environment.

### 2.3. Expression of Recombinant LstiCBP in E. Coliand Chitin-Binding Analysis

**Figure 4:**
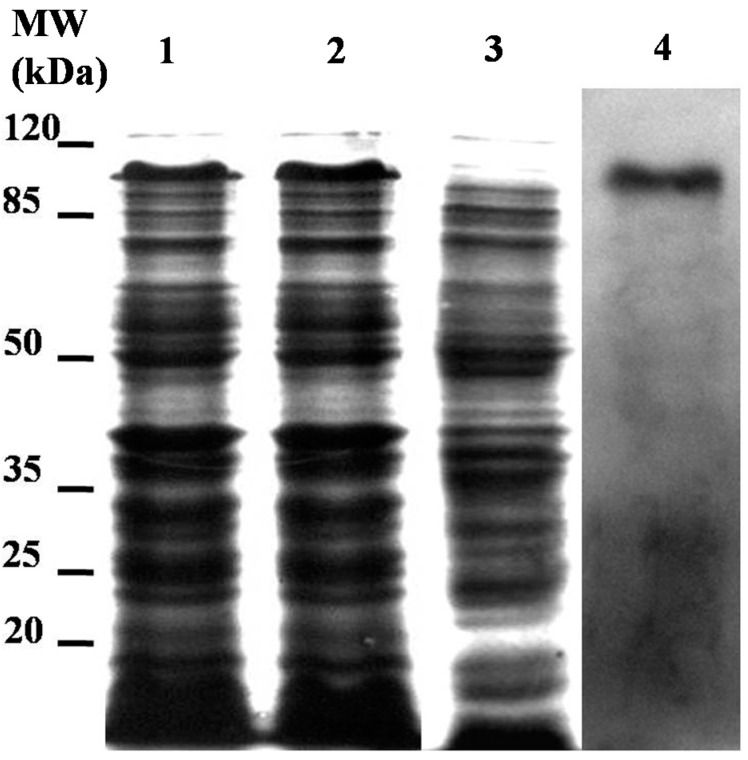
SDS-PAGE and Western blot analysis of expressed recombine LstiCBP. *M*w. Molecular weight marker; **1**. 1.0 mmol/L IPTG induced *E. coli* pET/LstiCBP; **2**. 2.0 mmol/L IPTG induced *E. coli* pET/LstiCBP; **3**. IPTG non-induced *E.coli* pET/LstiCBP; **4**. Western blot.

The LstiCBP ORF was amplified using primers CBP-MFP and CBP-MRP (Table 1) and inserted into the pET30 vector. The recombinant plasmid DNA was transformed into the *E. coli* BL21 strain, and its expression was induced. The cells were then frozen, thawed and disrupted by sonication. The cell lysates were electrophoresed on SDS-PAGE gels, and the lysates from the induced cells showed strong expression of the expected band (approximately 105 kD) (Figure 4), which implied that the recombinant LstiCBP was successfully expressed. To confirm that the expressed product was the expected recombinant protein, Western blot was performed using the anti-6 × His antibody. The induction of the transformed recombinant plasmid also resulted in a 105-kD band (Figure 4).

**Figure 5:**
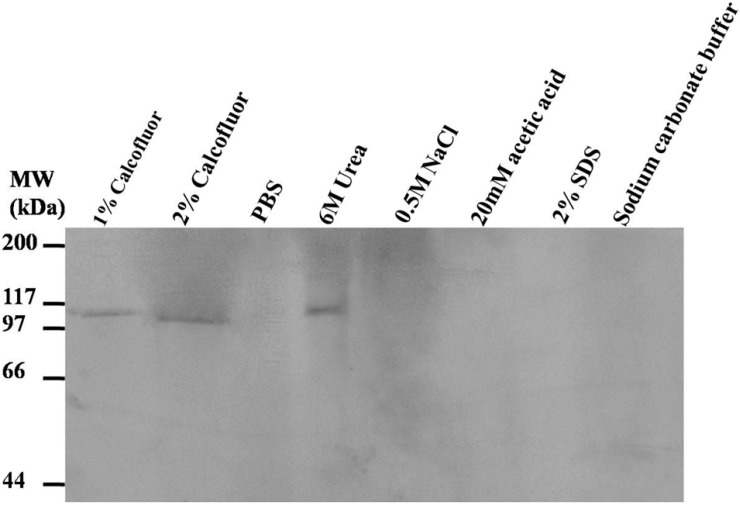
Analysis of chitin-binding activity of recombinant LstiCBP. LstiCBP treated with 1% Calcoflour, 2% Calcoflour, PBS, 6 M Urea, 0.5 M NaCl, 20 mM acetic acid, 2% SDS, Sodium carbonate buffer.

Most chitin-binding proteins contain one or more peritrophin domains, which are thought to be an important indication that the protein can bind to chitin. In this study, LstiCBP was successfully expressed in *E. coli*, and the recombinant protein exhibited obviously chitin-binding activity. Chitin-bound LstiCBP could only be released from the chitin by the competitive chitin-binding reagent Calcofluor (Sigma, St. Louis, MO, USA) or by the denaturing reagent urea (6 M). Treatments of the LstiCBP/chitin complex with PBS, 0.5 M NaCl, 2% SDS, 20 mM acetic acid or 0.1 M sodium carbonate buffer (pH 10.5) did not result in detectable dissociation of LstiCBP from chitin (Figure 5). *In vitro* chitin-binding analysis showed that the recombinant LstiCBP bound strongly to chitin, whereas only a strong denaturing reagent could release the recombinant proteins from chitin (Figure 5). Therefore, we conclude that LstiCBP is a Class 3 PM protein. The negative control didn’t show chitin-binding activity (result was not show).

### 2.4. Expression Profiles and Localization of LstiCBP

**Figure 6:**
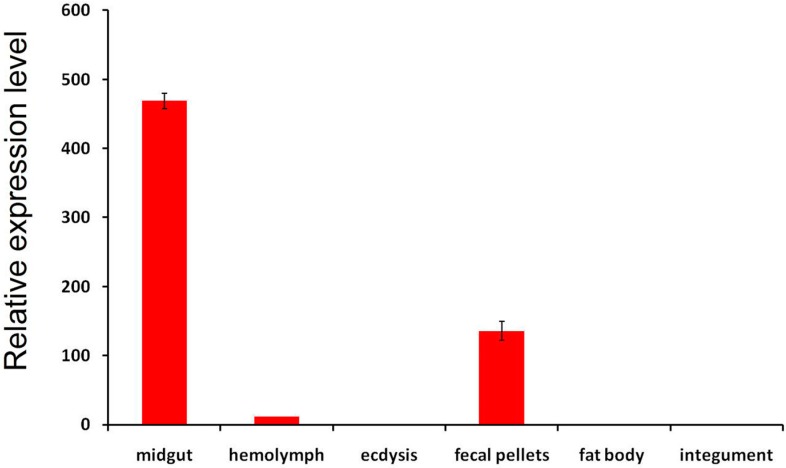
qPCR analysis of LstiCBP expressed in different tissues of *L. sticticalis* larvae. cDNAs were amplified with specific primers from midgut, hemolymph, ecdysis, fecal pellets, fat body and integument.

**Figure 7:**
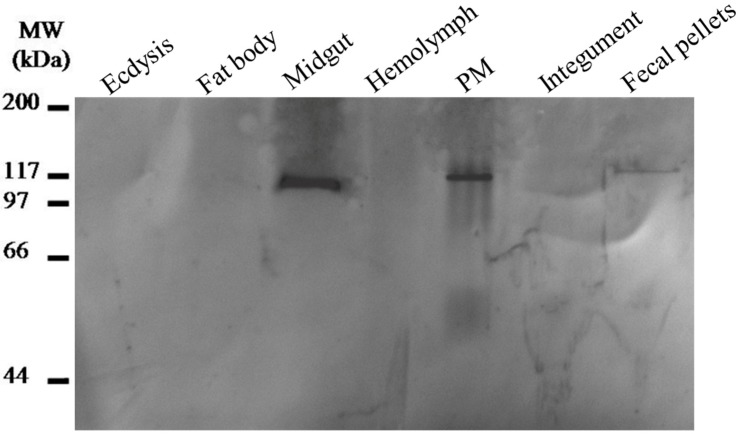
Identification of LstiCBP from *L. sticticalis* larval midgut proteins by Western blot analysis with antibodies specific to LstiCBP. Proteins were from the midgut, PM, hemolymph, ecdysis, fecal pellets, fat body and integument.

First, the expression of LstiCBP was studied using quantitative PCR. The midgut, head, hemolymph, ecdysis, fecal pellets, fat body and integument were dissected from larvae on day three of the fifth instar to use in this study. The desired product was largely amplified from cDNA templates that had been reverse-transcribed from total RNA from the midgut, with only a few products derived from other tissues, suggesting that LstiCBP is expressed specifically in the midgut (Figure 6). Then, LstiCBP was detected by Western blot using an antibody specific to LstiCBP. The result was consistent with the results from reverse transcription-PCR: the expected band was most strongly expressed in the midgut and PM, and the weaker band was detected in fecal pellets. No positive band was found in the ecdysis, fat body, hemolymph or integument (Figure 7), which suggests that the LstiCBP protein is synthesized in the midgut and transferred to the PM, where it is involved in maintaining the PM structure. LstiCBP is therefore a PM protein.

## 3. Experimental Section

### 3.1. Insect Larvae

A laboratory colony of *L. sticticalis* was established from larvae collected from the field in Zhangjiakou City (113°49'E, 42°28'N) of Inner Mongolia, China in 2009. The larvae were maintained at 22 ± 1 °C and 70%–80% RH and under a L:D 16:8 photoperiod until pupation and adult emergence. Adults were provided a 5% glucose solution (*w*/*v*) as supplemental food daily, and the larvae were reared in 500 mL beakers and fed daily with fresh leaves of *Chenopodium album* L. until they stopped feeding. The body parts to be used for analysis were isolated from 5th instar larvae and were stored at −70 °C until use.

### 3.2. Cloning and Sequencing of LstiCBP

A cDNA expression library of the *L. sticticalis* peritrophic midgut was screened by subtractive immunoscreening with antibodies against a collection of PM proteins according to the descriptions by Wang *et al.* [32] and Guo *et al.* [18]. Positive cDNA clones were sequenced using the dideoxynucleotide chain termination method (Takara Co., Dalian, China), and the sequences from 282 clones were submitted to BLAST analysis in GenBank. The sequence of clone 49–52 was similar to a portion of known CBP genes but lacked the 5' end. Then, the complete cDNA sequence of the clones of interest were obtained by 5' RACE using the 5'-Full RACE Kit Core Set Ver.2.0 (TAKARA) according to the manufacturer’s protocol. The 5' gene-specific primers were designed from the partial coding sequences of LstiCBP. The sequences of the primers are shown in Table 1.

**Table 1:** Oligonucleotide primers used in cDNA fragment cloning and RACE reactions for *LstiCBP*.

Primer Name	Primer Sequence (5'-3')	Position
CBP-R1	CATCGGGACAGGCTTTCCGTGGTT	687–710
CBP-R2	TCCTCGGTACTGGTATTCCGTGATTACAAA	991–1020
CBP-R3	CGGACCACTCACATCGGTTTGCTTC	198–222
CBP-R4	CATCGGGACAGGCTTTCCGTGGTT	687–710
5'RACE Outer Primer:	CATGGCTACATGCTGACAGCCTA	-
5'RACE Inner Primer:	CGCGGATCCACAGCCTACTGATGATCAGTCGATG	-

### 3.3. Expression Pattern of LstiCBP

The fifth instar larvae midguts, hemolymphs, ecdysis, fecal pellets, fat bodies and integuments were collected, immediately frozen in liquid nitrogen, and then stored at −80 °C until use. Total RNA was isolated from different tissues using Trizol reagent (Invitrogen, Carlsbad, CA, USA) in triplicate and then treated with gDNA Eraser to remove residual genomic DNA. The quality and concentration of the RNA was estimated by determining the A260/A280 ratios, and the samples were diluted to the same concentration (0.1 μg/μL) using DEPC-treated water. For RT-PCR, cDNA was synthesized using a first-strand cDNA synthesis kit (TaKaRa Co., Dalian, China). Taqman primers and probes were designed using Primer Express 3.0 (Applied Biosytems, Grand Island, NY, USA) and are listed in Table 2. The Taqman probes were labeled at their 5' ends with the FAM reporter dye and at their 3' ends with the quencher dye TAMRA. 18S rRNA was used as an endogenous control to normalize the results of variable target genes and to correct for sample-to-sample variation.

**Table 2:** Oligonucleotide primers used in qPCR.

Gene Name	Primer Name	Sequence (5'-3')
CBP	Forward	GCCCATGAAATATGTACCAAGTTCTAT
	Reverse	CAGTCGCACTCGTCTTTGGA
	Probe	FAM-ACGGCAAGCCTGTCGCCCTCA-TAMRA
18S rRNA	Forward	CAGGCTAGAGTCTCGTTCGTTACC
	Reverse	CAACACGGGAAATCTCACCAG
	Probe	FAM-CAAATCGCTCCACCAACTAAGAACGGC-TAMRA

Real-time qPCR was conducted using an ABI Prism 7500 Fast Detection System. Each amplification reaction was performed using a 20 μL reaction mixture under the following conditions: denaturation at 95 °C for 10 s followed by 40 cycles of 95 °C for 5 s, 60 °C for 34 s. Relative quantification was performed using the comparative 2^−ΔΔ*C*t^ method [35]. All data were normalized to endogenous18S rRNA levels from the same tissue samples, and the relative fold change in different tissues was calculated using the transcript level of the ecdysis as a calibrator. Thus, the relative fold change in different tissues was assessed by comparing the expression level of CBP in the tissues to that in the ecdysis.

### 3.4. Expression of Recombinant LstiCBP in E. coli and Chitin-Binding Analysis

To construct pET30-LstiCBP, the ORF of *LstiCBP* was amplified by PCR using primers CBP-MFP(5'-CG
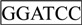
AAATCTGGGGATAGTGGTATAAAC-3') and CBP-MRP(5'-G
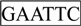
AAACCCATCGCATAAAAGTG-3') (the boxed sequences indicate the *Bam*H I and *Eco*R I restriction sites, respectively). The PCR product was inserted into the *Bam*H I and *Eco*R I sites of pET30, and transformed into the *E. coli* BL21 strain. After 3 h preincubation, the recombinant LstiCBP was induced by adding isopropyl-beta-d-thiogalactopyranoside (IPTG) at a final concentration of 2.0 mM for 4 h. The cells (1 L) were harvested by centrifugation, and the pellets were homogenized in phosphate-buffered saline (PBS, 0.04 M, pH 7.0). After centrifugation at 12,000× *g* for 20 min at 4 °C, the supernatants were dried and stored at −70 °C until use. Before SDS-PAGE, the cells were thawed and disrupted in PBS by sonication (5 s, 5 passes, 4 °C). The cell lysates from the cells before or after induction with IPTG were mixed with SDS-PAGE sample buffer, boiled for 10 min, centrifugation at 12,000× *g* for 10 min and supernatants were loaded onto an 8% SDS-PAGE gel. Western blot was performed following the description of Wei *et al.* [36]. After SDS-PAGE, the proteins were blotted onto a PVDF membrane (Hybond-P, Amersham, Zhengzhou, China), and the membrane was incubated with antibodies to 6 × His for 2 h at 37 °C. After washing in PBST (PBS-Tween, Sigma-aldrich, Shanghai, China), the membrane was incubated with secondary antibodies (HRP-conjugated goat anti-rabbit IgG, dilution 1/2000) for 2 h at 37 °C and then washed thoroughly with PBST. Antibody binding was detected using a DAB stock stain kit (Sino-American Biotechnology Co., Luoyang, China).

The chitin-binding activity of LstiCBP was analyzed using the chitin-binding assay described by Wang *et al.* [32]. First, regenerated chitin for the chitin-binding assay was prepared from chitosan (Sigma Corporation, St. Louis, MO, USA) by the method of Molano *et al.* [37]. One gram of chitosan is ground in a mortar while adding slowly and in small portions 20 mL of 10% acetic acid, and is allowed to stand overnight at room temperature. The next day, 90 mL of methanol are added slowly with mixing and the cloudy solution is filtered. The filtrate is placed in a beaker on a magnetic stirrer and 1.5 mL of acetic anhydride were added. After about 1 min, the mixture gels. The gel is allowed to stand for about 30 min and then is cut up into small pieces with a spatula. After covering with methanol the suspension is homogenized for 1 min at maximum speed. The finely dispersed chitin is filtered with a medium porosity sintered-glass funnel and is washed with water to neutrality. The chitin is resuspended in 0.02% sodium azide to a concentration of about 15 mg/mL. Then, recombinant LstiCBP was isolated by incubating 1 mL LstiCBP-containing cell culture medium with 40 mg (wet weight) of regenerated chitin to allow the LstiCBP protein to bind to chitin at 41 °C in suspension for 1 h in the presence of a protease inhibitor cocktail (0.5 mg/mL leupeptin, 1 mg/mL pepstatin, 1 mM EDTA and 1 mM phenylmethylsulfonyl fluoride). The regenerated chitin bound to LstiCBP was washed thoroughly with PBS followed by centrifugation. Aliquots of the resulting chitin-LstiCBP complexes were incubated with different solubilising conditions, PBS, 2% SDS, 6 M Urea, 1% Calcofluor, 2% Calcofluor, 0.5 M NaCl, 0.1 M NaHCO_3_-Na_2_CO_3_ buffer (pH 10.5) or 20 mM acetic acid. After 15 min incubation at room temperature, the supernatants containing the LstiCBP protein released from the chitin were collected by centrifugation and analyzed by SDS-PAGE analysis. IPTG induced *E. coli* BL21 strain with pET30 was taken as the negative control protein that treated as above.

### 3.5. Preparation of Antibodies that React to LstiCBP

An antibody specific for LstiCBP was prepared from an antiserum made against a collection of all *L. sticticalis* midgut PM proteins. The recombinant LstiCBP protein was immobilized onto a piece of supported nitrocellulose membrane (Optitran BA-S85, Schleicher & Schuell, Keene, NH, USA) by incubating the membrane with the recombinant protein at room temperature for 1 h, followed by extensive washing (5 times) with phosphate-buffered saline (PBS) and incubation in 3% bovine serum albumin (BSA) for 3 h. The nitrocellulose membrane was then incubated with a 100-fold dilution of the *S. exigua* PM protein polyclonal antiserum in PBS with 3% BSA at room temperature for 3 h or at 4 °C overnight to allow the antibodies in the antiserum to bind to the blotted membrane. Then, the membrane was thoroughly washed five times with PBS, and the antibodies specifically bound to Lsti99 were eluted from the membrane by incubation in 5 mL of 0.1 M glycine buffer (pH 2.5) at room temperature for 10 min, followed by addition of 0.5 mL of 1 M Tris-HCl buffer (pH 8.0) to neutralize the pH of the antibody preparation [38].

### 3.6. Western Blot Analysis

The proteins used in Western blot were extracted from tissues by homogenization in PBS and centrifugation at 12,000× *g* for 20 min at 4 °C. The supernatants were then dried and stored at −70 °C. The protein extracts were mixed with SDS-PAGE sample buffer and then boiled for 10 min and immediately loaded onto a 12% SDS-PAGE gel. After SDS-PAGE, the proteins were blotted onto a PVDF membrane (Hybond-P, Amersham, Zhengzhou, China), and the membrane was incubated with LstiCBP antibodies for 2 h at 37 °C. After washing in PBST (PBS-Tween, Sigma-aldrich, Shanghai, China), the membrane was incubated with secondary antibodies (HRP-conjugated goat anti-rabbit IgG, dilution 1/2000) for 2 h at 37 °C and thoroughly washed in PBST. Antibody binding was detected using a DAB stock stain kit (Sino-American Biotechnology Co., Luoyang, China) [36].

## 4. Conclusions

A novel midgut peritrophic membrane (PM) protein, LstiCBP, from *Loxostege sticticalis* was identified. The results from this study show that LstiCBP contains 8 peritrophin-A domains and has chitin-binding activity, which is similar to the currently known peritrophin-type PM proteins. Furthermore, LstiCBP is strongly associated with the PM and is mainly expressed in the midgut. Until recently, the molecular structure, function and mechanism of the PM have been unclear. Our description of LstiCBP expands the knowledge of the insect PM. However, as a new protein that was first found in *L*. *sticticalis*, the biochemical and physiological functions of this protein must be studied further.
